# Screening sesame (*Sesamum indicum*) for resistance to multiple root-knot nematode species (*Meloidogyne* spp.)

**DOI:** 10.2478/jofnem-2025-0017

**Published:** 2025-04-12

**Authors:** Marcela Chávez, Adrienne Gorny, Angela Post, David Suchoff

**Affiliations:** Department of Crop and Soil Sciences, North Carolina State University, Raleigh, NC 27695, USA; Department of Entomology and Plant Pathology, North Carolina State University, Raleigh, NC 27695, USA

**Keywords:** sesame, greenhouse, guava root-knot nematode, southern root-knot nematode, northern root-knot nematode, peanut root-knot nematode, reproductive factor

## Abstract

Root-knot nematodes (RKN; *Meloidogyne* spp.) are among the most damaging plant-parasitic nematodes. They parasitize almost every species of higher plant and induce the formation of galls along the plant roots, which are detrimental to plant growth. North Carolina’s leading field crops are sweetpotato (*Ipomoea batatas* (L.) Lam.), soybean (*Glycine max* L. Merr), cotton (*Gossypium hirsutum* L.), and tobacco (*Nicotiana tabacum* L.), which are all hosts to several root-knot nematode species. This pathogen represents a major threat to farmers, obligating them to seek alternative crops that are non-host to root-knot nematodes that will help decrease soil populations and provide economic revenue. We tested seven sesame cultivars for their host status and potential resistance to four *Meloidogyne* species (*M. arenaria*, *M. incognita*, *M. enterolobii*, and *M. hapla*). We inoculated sesame seedlings with 1,000 nematode eggs of each species. Sixty days after inoculation, we harvested the plants to evaluate a visual gall severity rating, measure final egg counts, and calculate the reproductive factor (RF). All sesame cultivars had a significantly lower RF than the tomato (*Solanum lycopersicum* L.) cv. Rutgers control for all species of RKN except *M. arenaria*. The RF values for sesame cultivars inoculated with *M. incognita* and *M. hapla* were not significantly different from one another; however, there were significant differences in RF among sesame cultivars inoculated with *M. enterolobii*, suggesting that genetic variability of the host may play an important role in host status and conferring resistance.

## Introduction

Root-knot nematodes (*Meloidogyne* spp.; RKN) are among the most damaging groups of plant-parasitic nematodes. They parasitize almost every species of higher plant by penetrating the host’s roots and inducing the formation of galls or knot-like swellings along the plant roots (Gill & McSorley, 2011; Moens et al., 2009). These galls are detrimental to plant growth since they interfere with the crop’s ability to absorb water and nutrients (Khan et al., 2017). Additionally, their feeding can cause early senescence, wilting, premature defoliation, stunting, and total crop loss (Khan et al., 2017; Moens et al., 2009; Perry & Moens, 2011). Under ideal environmental conditions and depending on the RKN species and host species, the life cycle of most RKN is completed in 3–6 weeks (Subbotin et al., 2021). This allows for several generations to develop during one cropping season, which can lead to a rapid increase in population and severe crop damage (Moens et al., 2009). The major species of RKN reproduce by parthenogenesis, where the progeny are “clones” of the female adult; the production of males is lower, and they do not have a sexual role (Bert et al., 2011).

Historically, RKN management has been achieved with chemical nematicides, yet the extensive use of these chemicals poses a potential hazard to the environment and human health, which has led to a decline or ban of many products (Noling & Becker, 1994; Perry & Moens, 2011). Therefore, development and implementation of economically feasible and environmentally safe alternatives is needed (Fernández et al., 2001; Noling & Becker, 1994). Crop rotation systems may introduce resistant or poor hosts that may either inhibit pathogen growth through failing to meet the nutritional needs of the pathogen, producing toxic compounds detrimental to the pathogen, or supporting antagonistic microbial communities in the rhizosphere (Rodriguez-Kabana & Canullo, 1992; Travedi & Barker, 1986). The magnitude of nematode control attained through crop rotation will vary with the year, cultivar, and resistance of the plant species, associated weed hosts, environmental conditions, and the length of the rotation (Travedi & Barker, 1986).

The introduction of sesame (*Sesamum indicum*) into a cropping system has proven to reduce RKN levels (Couch et al., 2017). The nematicidal effect of sesame is attributed to the lignans sesamin and sesamolin, which are natural antioxidants found only in this crop (Ashri, 1998; Bedigian & Harlan, 1986; Couch et al., 2017). Sesame is a relatively new crop in the US, with its production centered in Texas, Oklahoma, Arizona, and Kansas (Grichar et al., 2011; Gloaguen et al., 2018; Langham, 2007).

There is a need and interest to explore other suitable areas for sesame production, particularly the southeast United States due to its warm, long growing season and greater annual precipitation compared to the Southwest (Couch et al., 2017; Gloaguen et al., 2018). Nematode management is critical in North Carolina (NC), where major crops such as sweetpotato (*Ipomoea batatas* (L.) Lam), cotton (*Gossypium hirsutum* L.), tobacco (*Nicotiana tabacum* L.), and soybean (*Glycine max* L. Merr) are susceptible to various RKN species, including the southern root-knot (*M. incognita*), northern root-knot (*M. hapla*), peanut root-knot (*M. arenaria*), and the guava root-knot (*M. enterolobii*) (Thiessen & Rivera, 2019; Quesada, 2018). While these RKN species pose a threat to crop production in NC, considerable attention has been given to guava root-knot nematode in recent years as it is relatively new and causes significant damage to the state’s sweetpotato industry (Schwarz et al., 2021; Ye et al., 2013). North Carolina is the largest sweetpotato producing state, with 32,375 ha with a production value of $225,005,000 in 2022 (USDA, 2022). The guava root-knot nematode causes severe yield loss by affecting root quality and tonnage, thus reducing the overall marketable value of the produce (Brito et al., 2020; Moens et al., 2009).

With the introduction of sesame into North Carolina’s agronomic rotations, it is necessary to evaluate the crop’s potential to suppress nematode populations utilizing the cultivars proposed for this region. The objectives of this study were to evaluate sesame resistance to *M. incognita*, *M. hapla*, *M. arenaria*, and *M. enterolobii*, as well as to identify which sesame cultivars may provide higher resistance.

## Materials and Methods

Nematode resistance trials were conducted at the North Carolina State University Method Road Greenhouse Complex, in Raleigh, NC. Seven commercially available sesame cultivars (Table 1) were screened for resistance to four RKN species) including *Meloidogyne incognita*, *M. arenaria*, *M. hapla*, and *M. enterolobii*. Seeds were provided by Equinom Ltd. (Givat Brenner, Israel) and Sesaco Corporation (Austin TX, USA). The tomato (*Solanum lycopersicum* L.) cv. Rutgers was used as a susceptible control. Three sesame seeds were planted in a 3.8 cm diameter × 20 cm deep (approximately 76 cm^3^ in volume) plastic cone container filled with a steam-sterilized 1:1 (v:v) sand-to-soil mixture, with a composition of 88% sand, 9% clay, 3% silt, and 0.20% organic matter. Seven days after planting (DAP), each container was left with one emerged seedling by carefully removing any other emerged seedlings. Each container was fertilized with 5 g of slow-release, balanced fertilizer (14-14-14 Osmocote Smart-Release Plant Food, The Scotts Company, Marysville, OH). The greenhouse was maintained at 25 °C to 28 °C, and plants were watered twice a week in the first 49 DAP and once a week from day 50 to harvest.

**Table 1:** Sesame cultivars screened for resistance to *Meloidogyne incognita*, *M. hapla*, *M. arenaria*, and *M. enterolobii* in a two greenhouse pot experiments.

**Company**	**Cultivar**
Equinom^x^	ES103
Equinom	ES107
Equinom	ES108
Sesaco^y^	S39
Sesaco	S3301
Sesaco	S3251
Sesaco	S3276

x Equinom Ltd. (Givat Brenner, Israel).

y Sesaco Corporation (Austin TX, USA).

Thirty DAP, containers were inoculated with 1,000 RKN eggs by pipetting the egg solution into a 1.5 cm deep hole made into the soil at the base of each plant, which allowed the sesame plant to develop its root system. Eggs were collected from the four RKN species individually from cultures maintained on ‘Rutgers’ tomato following the NaOCl extraction method described by Hussey and Barker (1973). Species identity of each RKN isolate is confirmed yearly through species-specific PCR primers and conducting the North Carolina Differential Host Test. To produce the initial inoculum, roots from each infected tomato were soaked in a 10% bleach solution for 40 seconds, gently massaging egg masses from the roots. The solution was then poured through a set of sieves (from top to bottom, 250 μm, 75 μm, 25 μm) and rinsed with water to remove residual bleach from the eggs. Eggs were rinsed to the bottom 25 μm sieve with water, then collected in a 50 mL centrifuge tube to a final volume of 35 mL with water. To ensure a clean sample to aid in rapid counting of inoculum density, a sucrose centrifugation step was included. Each tube was topped to 50 mL with a 70% sucrose solution and centrifuged at 400 *g* for 5 min. After centrifugation, the supernatant was sieved (25 μm) a second time and transferred into a clean 50 mL tube. Eggs were quantified by taking three 100 μl subsamples and counting the eggs using an inverted microscope (Nikon TMS, Nikon Instruments Inc., Melville, NY, USA) at 40x magnification. Egg concentrations were calculated and expressed per mL solution.

**Figure 1:**
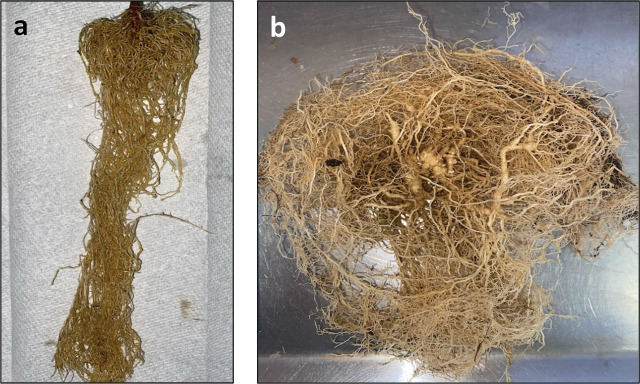
Plants were assessed for root gall severity following a 0–10 scale adapted from Zeck (1971). (a) example of a root gall severity score of 0 in a sesame root system and (b) root gall severity score of 5 in a ‘Rutgers’ tomato root system.

Sixty days post inoculation, the sesame and tomato control plants were cut at the crown to separate the roots. Each root system was rinsed with cold water to remove soil and fresh root weight was recorded. The trial was conducted twice and arranged in a complete randomized design with six replicate plants per cultivar × RKN species combination per trial. Four plants from each treatment group were given a visual root gall severity score based on the percentage of the root system galled using a scale of 0–10, with 0 representing 0% root galling, 6 representing 50% and 10 representing 100% (Zeck, 1971; Figure 1). RKN eggs were extracted from the same four individual root systems using the NaOCl method as described above. Three 1 mL subsamples were taken from each sample to quantify egg count. The average of these three subsamples was multiplied by the final extraction volume to estimate the total number of eggs per root system (final population, Pf).

Reproductive factor (RF) was calculated for each sample using a ratio of the final population (Pf) and the initial population (Pi; herein, 1,000). Reproductive factor values were used to determine the susceptibility or resistance of the sesame cultivars to the RKN species. An RF value less than 1.0 indicates a poor host or non-host resistance, while values greater than 1.0 indicate susceptibility (Ferris et al., 1993). An RF value equal to one indicates a maintenance host, in which the population of nematodes is neither increasing nor decreasing.

### Statistical analysis

Data from both trials were combined, and the trial was treated as a random effect. The effect of sesame cultivar was treated as fixed and results for each RKN species analyzed separately. Data were analyzed using the GLIMMIX procedure in SAS version 9.4 (SAS Institute, Cary, NC) and inspected for any violations against the assumptions of ANOVA. No violations (e.g., heteroskedasticity) were observed and thus the data were analyzed without transformation or the use of alternative distributions. When appropriate, Tukey’s HSD means separation test was employed at a significance of α = 0.05.

## Results and Discussion

### Root gall severity

There were significant differences in visual gall severity rating between all sesame cultivars and the tomato control (Table 2; *P* < 0.0001). Gall severity rating was significantly higher in the tomato control for all nematode species when compared to all the sesame cultivars. Among tomato control plants, the highest visual root gall score was observed in plants inoculated with *M. enterolobii* (6.3). Conversely, the lowest root gall score was observed from the tomato control inoculated with *M. hapla* (2.0). Root gall severity scores for sesame inoculated with *M. incognita* ranged from 0.0 to 0.1; with *M. hapla* from 0.0 to 0.1; with *M. arenaria* from 0.1 to 1.0; and with *M. enterolobii* from 0.3 to 1.4 (Table 2). Sesame root cells secrete chemical metabolites that may be toxic to nematodes and inhibit penetration and motility, which ultimately has a negative effect on population density, hence the lack of large visible root galls (Araya & Caswell-Chen, 1994; Tanda et al., 1988). Yet, some minute root galls were observed on some of the sesame varieties in this study, indicating a low level of reproduction.

**Table 2:** Visual root gall severity scores for sesame cultivars inoculated with 1,000 nematode eggs sixty days after inoculation. Values in the table are the mean of eight replicate plants. Root gall severity was rated on a scale of 0 to 10, with 0 = no root galling, 6 = approximately 50% of the root system galled, and 10 = 100% of the root system galled (adapted from Zeck 1971).

**Cultivar**	***Meloidogyne* Species**			
	***M. arenaria***	***M. enterolobii***	***M. hapla***	***M. incognita***
ES103	0.4 b	0.6 b^Z^	0.0 b	0.0 b
ES107	0.1 b	1.0 b	0.0 b	0.0 b
ES108	1.0 b	0.7 b	0.0 b	0.1 b
S39	0.1 b	0.8 b	0.0 b	0.0 b
S3276	0.4 b	0.9 b	0.1 b	0.0 b
S3251	0.6 b	0.3 b	0.0 b	0.0 b
S3301	0.7 b	1.4 b	0.0 b	0.1 b
Tomato cv. ‘Rutgers’	2.6 a	6.3 a	2.0 a	5.0 a
*P*-value	<0.0001	<0.0001	<0.0001	<0.0001

Z Means sharing a common letter within the column are not significantly different (*P* > 0.05).

### Reproductive factor

There were significant differences in RF among sesame cultivars inoculated with *M. hapla*, *M. enterolobii,* and *M. incognita* (*P* < 0.0001; Table 3). The RFs for the tomato control were 3.72 and 27.50 for *M. hapla* and *M. incognita*, respectively. There were significant differences in RF among sesame cultivars and the tomato control inoculated with *M. enterolobii*. Cultivar ES103 had the lowest RF (0.15) but was not statistically different from cultivars S3301, ES107, and ES108. Cultivar S39 had the highest RF (1.63) among all sesame cultivars but was not statistically different from cultivars S3276 and S3251. This indicates that resistance to *M. enterolobii* may be genetically controlled, and loci responsible for resistance to *M. enterolobii* may not be present in all commercial sesame cultivars. Although numerically different, there were no statistically significant differences in RF values for sesame cultivars or tomato control when inoculated with *M. arenaria* (*P* = 0.0649).

**Table 3:** Reproductive factor for sesame cultivars inoculated with 1,000 nematode eggs. Values in the table are the mean of eight replicate plants.

**Cultivar**	***Meloidogyne* Species**			
	***M. arenaria***	***M. enterolobii***	***M. hapla***	***M. incognita***

	**Reproductive factor (Pf/Pi)^Y^**			
ES103	0.68	0.15 a^Z^	0.12 a	0.04 a
S3301	1.01	0.43 ab	0.12 a	0.23 a
ES107	0.31	0.47 ab	1.05 a	0.03 a
ES108	0.81	0.16 ab	0.07 a	0.13 a
S3276	1.28	0.71 bc	0.69 a	0.08 a
S3251	0.44	0.75 bc	0.04 a	0.20 a
S39	1.13	1.63 c	0.18 a	0.04 a
Tomato cv. ‘Rutgers’	3.96	6.74 d	3.72 b	27.50 b
*P*-value	0.0649	<0.0001	<0.0001	<0.0001

Z Means sharing a common letter within species are not significantly different (*p* > 0.05)

Y Reproductive factor (RF) is the final nematode population divided by the initial population (herein, 1,000), evaluated at 60 days after inoculation. RF values < 1 indicate a poor host or non-host resistance, while values > 1 indicate susceptibility.

The results obtained in this study are comparable to those found in the literature, where sesame has been shown to suppress and lower RKN populations (Araya & Caswell-Chen, 1994; El-Nagdi & Youssef, 2015; Starr & Black, 1995). Starr and Black (1995) tested 10 sesame varieties in a pot experiment and found no reproduction of *M. incognita* in all but two varieties. Additionally, the sesame cultivars used in this study are different from any in the literature available, suggesting that sesame is broadly a poor-to non-host to many RKN species, regardless of the cultivar. The reproductive factor was significantly lower for all sesame cultivars compared to the control, except for *M. arenaria*. All sesame cultivars supported a low reproductive factor for *M enterolobii*, *M. hapla*, and *M. incognita*, which remained below the threshold for non-host resistance, with a few exceptions. Since there are significant differences among sesame cultivars inoculating *M. enterolobii*, we can infer that relative host status and resistance may be genetically linked. The suppressive effect of sesame on RKN populations under field conditions should be explored, especially in fields infested with *M. enterolobii*. It is unlikely that a one-year rotation with sesame will completely eradicate nematodes from heavily infested fields or eliminate the need for nematicides altogether; however, knowledge of the poor-host status of many sesame cultivars to several RKN species adds another powerful tool to the integrated pest management toolbox, and ultimately improve farmer economics and sustainability. We would recommend the use of cultivar ES103 and ES108 for farmers struggling with *M. enterolobii*, *M. hapla*, and *M. incognita*, since the data showed low RF values for these species. Additionally, we would recommend the use of other cultivars such as S39 and S3276, which produced low RF values when inoculated with *M. incognita*. Overall, the use of sesame, regardless of the cultivar, produced low RF values in three of the four nematode species tested in this study. We would recommend the use of sesame to farmers seeking to reduce *M. enterolobii*, *M. hapla*, and *M. incognita* populations.
